# A multimodal imaging–based integrative framework for HIV-associated cognitive impairment and treatment response

**DOI:** 10.3389/fnins.2026.1775047

**Published:** 2026-04-13

**Authors:** Xing Qiu, Md Nasir Uddin, Lu Wang, Alicia Tyrell, Abrar Faiyaz, Nhat Hoang, Yuchuan Zhuang, Madalina E. Tivarus, Jianhui Zhong, Miriam T. Weber, Giovanni Schifitto

**Affiliations:** 1 Department of Biostatistics and Computational Biology, University of Rochester, Rochester, NY, United States; 2 Department of Neurology, University of Rochester, Rochester, NY, United States; 3 Department of Biomedical Engineering, University of Rochester, Rochester, NY, United States; 4 Department of Electrical and Computer Engineering, University of Rochester, Rochester, NY, United States; 5 Department of Physics and Astronomy, University of Rochester, Rochester, NY, United States; 6 Department of Imaging Sciences, University of Rochester, Rochester, NY, United States; 7 Department of Neuroscience, University of Rochester, Rochester, NY, United States; 8 Department of Obstetrics and Gynecology, University of Rochester, Rochester, NY, United States

**Keywords:** brain, cognitive impairment, HIV, interpretable machine learning, MRI, multimodal integration, neuroimaging

## Abstract

**Introduction:**

Combination antiretroviral therapy (cART) has been shown to reduce inflammation in persons with HIV (PWH), leading to overall improvements in cognition. However, these improvements are patient-dependent and not always observable over short treatment periods.

**Methods:**

We applied a multimodal integrative model to associate various baseline MR neuroimaging metrics with baseline neurocognitive performance and their longitudinal changes over 12 weeks of cART treatment. Features in our model included volumetric data, cerebral blood flow metrics, cerebrovascular reactivity, and diffusion MRI data from cortical, subcortical, and white matter regions of the brain. Our integrative model, which includes multilayered principal component analysis, penalized regression, and feature weight back-propagation, is designed for “large p, small n” data and offers better interpretability than deep-learning methods.

**Results:**

There is a modest association between imaging metrics and baseline neurocognitive scores for both PWH and age-matched healthy controls, driven primarily by subcortical regions. In contrast, baseline imaging features exhibited stronger associations with longitudinal changes in cognitive performance over 12 weeks of cART in PWH than with baseline cognitive scores. The multimodal integrative model outperformed all comparable unimodal models in explaining longitudinal cognitive change. Among unimodal analyses, models based on cerebral blood flow and free-water-corrected fractional anisotropy demonstrated the strongest associations. Frequently selected predictors included the frontal pole (cortical gray matter); the amygdala, putamen, and hippocampus (subcortical gray matter); and the posterior limb of the internal capsule (white matter).

**Discussion:**

Our approach provides an interpretable statistical framework that integrates complementary information across various imaging modalities into a robust and interpretable model for short-term cognitive trajectories in PWH undergoing cART.

## 1 Introduction

The introduction of combination antiretroviral therapy (cART) has revolutionized the management of HIV infection, significantly reducing morbidity and mortality. One of the critical benefits of cART is its ability to reduce neuroinflammation, which has been linked to improvements in cognition among people with HIV (PWH). Despite these advancements, studies conducted by our team and others showed that cognitive improvements under cART are not uniformly observed across all patients, with some individuals showing significant gains and others exhibiting minimal or no improvement, over relatively short treatment periods (Cysique et al., 2009, Heaton et al., 2015, Gates et al., 2016, Uddin et al., 2021, Weber et al., 2022, Singh et al., 2023). This variability motivated us to seek a deeper understanding of the mechanisms underlying cognitive changes in PWH during cART initiation.

Neurocognitive impairment remains a significant concern for PWH (Heaton et al., 2010), affecting their quality of life and function. Neurocognitive deficits in PWH can be attributed to various factors, including direct effects from viral proteins released by glia cells, chronic neuroinflammation from activated glia cells (Jones and Power, 2006, Killingsworth and Spudich, 2022), and vascular co-morbid conditions (Chow et al., 2012, Chow et al., 2016). These effects eventually lead to brain structural and functional changes, which can be evaluated by various brain imaging markers. Noninvasive brain imaging techniques, such as magnetic resonance imaging (MRI), have been instrumental in identifying structural and functional abnormalities associated with HIV infection. In recent years, studies conducted by our team and others have demonstrated that multiple MRI techniques offer distinct perspectives in assessing differences between PWH and healthy controls, as well as monitoring the recovery in PWH undergoing cART treatment (Chang et al., 1999, Ragin et al., 2004, Strain et al., 2017, Zhuang et al., 2017, Murray et al., 2020, Uddin et al., 2021, Zhuang et al., 2021, Weber et al., 2022, Singh et al., 2023, Uddin et al., 2024).

PWH treated with cART show, as a group, stable cognitive performance over several years, but continue to have worse cognitive performance compared to healthy controls. Neuroimaging differences also continue to persist when comparing these two groups. In this regard, cART naïve PWH are more likely, at least in the short term, to show cognitive improvement and changes in neuroimaging markers (Zhuang et al., 2017, Weber et al., 2022). However, which specific imaging modality or combination of modalities is best to predict cognitive changes remains to be determined. This is partly because most individual markers from a single imaging modality may show weak associations with the outcome variable. It is a standard clinical practice to use multiple imaging modalities to assess the presence of brain pathology. It is therefore natural to consider a multimodal statistical model to integrate various types of imaging metrics and develop a strong predictor for the outcome variable. However, integrating multimodal imaging data faces two main challenges. First, compared with a typical unimodality study, it takes more resources and broader expertise to collect and process brain imaging data across multiple modalities within a single clinical study. Secondly, it is also statistically challenging to train a reliable and interpretable multimodality statistical model in an under-determined (“large p, small n”) situation. For example, cross-validation (CV), a standard validation technique in machine learning, are known to be highly unstable in small sample settings (Hawkins et al., 2003, Varoquaux, 2018) and may lead to “optimizer’s curse,” in which the validation performance is significantly over-optimistic compared to true test set error (Smith and Winkler, 2006, Gupta and Rusmevichientong, 2021, Cory-Wright and Gómez, 2025). In this study, we introduce a multimodal integrative model that combines feature screening, multi-layered principal component analysis (PCA), and regression with AIC-based model selection to rigorously control model complexity. A key contribution is that we developed an equivalent weight back-propagation algorithm that translates a complex predictive model based on multi-layered PCA back to the original feature space, which produces standard-deviation-adjusted weights for users to rank and directly interpret the original imaging features. This level of biological interpretability is often lacking from “black-box” style machine learning approaches. Using this approach, we studied the associations between baseline brain imaging metrics and both baseline neurocognitive scores and their changes over a 12-week period from cART treatment initiation.

Our investigation focuses on the following MRI modalities: T1-weighted MRI derived subcortical volumes (Vol), cortical thickness (CT), fractal dimensionality (FD); diffusion MRI derived extracellular free water (FW), fractional anisotropy (FA), mean diffusivity (MD); arterial spin labeling (ASL) derived cerebral blood flow (CBF); resting-state fMRI based cerebrovascular reactivity (CVR). Those measurements are collected from various regions of interest (ROIs) that can be divided into three key brain areas: cortical gray matter, subcortical gray matter, and white matter. We hypothesized that our multimodal integrative model would outperform unimodal models in predicting longitudinal changes in neurocognitive scores, providing a robust tool for understanding and potentially forecasting cognitive trajectories in this population.

## 2 Materials and methods

### 2.1 Research subjects

Forty-five treatment-naïve PWH and 92 HIV uninfected (HIV–) participants were enrolled in a study assessing the potential neurotoxicity of combination antiretroviral therapy treatment (cART) at the University of Rochester Medical Center. Of them, 30 PWH and 56 controls had complete multimodality data, and they comprise the 86 subjects analyzed in this study. It should be noted that the age and sex of the PWH cART-naïve individuals recruited reflected those of individuals infected who were receiving care during the study period. All participants provided written informed consent before enrollment according to the institutional protocol and underwent clinical, laboratory, and brain MRI exams. All experiments were performed in accordance with relevant guidelines and regulations. The details of the study have been provided elsewhere (Weber et al., 2022).

Data used in this study include: (a) various types of MRI metrics in PWH, cART naïve collected at the baseline (prior to starting cART) and after 12 weeks of cART treatment, (b) neuropsychological assessments measured at both the baseline and after 12 weeks of cART treatment in PWH. These two visits were chosen to represent the clearest difference in cognitive performance within the PWH group. Demographic information about study participants is provided in Table 1; more details can be found in Table 1 of Weber et al. (2022).

**TABLE 1:** Participant characteristics, stratified by the HIV status.

Variable	Control	PWH
	*N* = 56	*N* = 30
Age, years, mean ± SD	41.3 ± 15.0	33.3 ± 13.1
Gender, n (%)		
Female	24 (42.9%)	2 (6.67%)
Male	32 (57.1%)	28 (93.3%)
Ethnicity, n (%):		
Hispanic or Latino	2 (3.57%)	3 (10.0%)
Not Hispanic or Latino	54 (96.4%)	27 (90.0%)
Race, n (%)		
Black	5 (8.93%)	14 (46.7%)
Other	4 (7.14%)	0 (0.00%)
White	47 (83.9%)	16 (53.3%)
Bachelor’s degree, n (%)		
Yes	40 (71.4%)	9 (30.0%)
No	16 (28.6%)	21 (70.0%)

### 2.2 Data acquisition

#### 2.2.1 Neuropsychological assessments

The neurocognitive evaluation was performed by staff trained and supervised by a clinical neuropsychologist. Tests of Executive Function (Trailmaking Test Parts A and B, Stroop Interference task), Speed of Information Processing (Symbol Digit Modalities Test and Stroop 2 Color Naming), Attention and Working Memory [CalCAP(CRT4) and WAIS-III Letter-Number Sequencing], Learning [Rey Auditory Verbal Learning Test AVLT (trials 1–5), Rey Complex Figure Test Immediate Recall], Memory (Rey Auditory Verbal Learning Test RAVLT Delayed Recall, Rey Complex Figure Test Delayed Recall), and Motor (Grooved Pegboard, left and right hand) were administered at each visit. Premorbid intellectual functioning ability was estimated via WRAT-4 Reading at the baseline visit only. Raw scores were converted to z-scores using test manual norms, but the z-scores were cut off at ± 3 standard deviations (SD) above and below the mean values. Cognitive domain scores were created by averaging the z-scores of tests within each domain. A total summary score was calculated by summing the z-scores of the six cognitive domains measured (Executive Function, Speed of Information Processing, Attention and Working Memory, Learning, Memory, and Motor).

#### 2.2.2 Magnetic resonance imaging

MRI was performed on 3T scanner (MAGNETOM Trio, Siemens, Erlangen, Germany) equipped with 32-channel head coil.

##### 2.2.2.1 Anatomical imaging

The T1-weighted (T1w) images were acquired using a 3D magnetization prepared rapid acquisition gradient-echo (MPRAGE) sequence with inversion time (TI) = 1,100 ms, repetition time (TR) = 2,530 ms; echo time (TE) = 3.44 ms; flip angle = 7°; field of view (FOV) = 256 × 256; GRAPPA = 2; number of slices = 192; voxel size = 1.0 × 1.0 × 1.0 mm^3^.

##### 2.2.2.2 Diffusion tensor imaging

Diffusion weighted images (DWI) were acquired using a single shot spin echo echo-planar imaging (SE-EPI) sequence with the following scan parameters: 60 diffusion-encoded images (*b* = 1,000 s/mm^2^), 10 non-diffusion weighted reference images (*b* = 0 s/mm2); TR = 8,900 ms; TE = 86 ms; FOV = 256 × 256; GRAPPA = 2; number of slices = 70; voxel size = 2.0 × 2.0 × 2.0 mm^3^. In order to correct for EPI distortions, a double-echo gradient echo field map sequence was also acquired (TR = 701 ms; TE1 = 5.19 ms; TE2 = 7.65 ms; FOV = 256 × 256; flip angle = 60°; number of slices = 70; voxel size = 2.0 × 2.0 × 2.0 mm^3^).

##### 2.2.2.3 Resting-state fMRI (rs-fMRI)

The rs-fMRI scans were acquired using a gradient echo-echo planar imaging (GE-EPI) sequence with parameters TR = 2,000 ms, TE = 30 ms, 150 volumes, and voxel size = 4.0 × 4.0 × 4.0 mm^3^. Participants were instructed to keep their eyes closed throughout the scan.

##### 2.2.2.4 Perfusion imaging

A pseudo-continuous arterial spin labeling (pCASL) sequence was used to acquire perfusion MRI images with scan parameters—TR = 3,530, TE = 22.62 ms and voxel size = 3.8 × 3.8 × 5 mm^3^. A tag-control pair technique with 36 averages was used, incorporating a labeling duration of 1.5 s and a single post-label delay (PLD) of 1.5 s. Additionally, the equilibrium magnetization of arterial blood (M0) image was acquired with one average using a TR of 5,000 ms.

### 2.3 Data analysis

Image analyses were conducted using multiple image processing tools, including FMRIB’s Software Library (Jenkinson et al., 2012) (FSL),^1^ ANTs (Avants et al., 2009),^2^ FreeSurfer (Fischl, 2012),^3^ and MATLAB (version 2022b; MathWorks, Natick, MA). All MRI images were visually inspected for any severe artifacts including motion and signal dropout.

#### 2.3.1 Anatomical imaging

T1-weighted images were reoriented, cropped, bias-field corrected, skull-stripped, registered both linearly and nonlinearly to MNI152 2 mm standard space, and segmented by tissue type using FSL’s (version 5.11) anatomical processing script, fsl_anat (Smith et al., 2004, Woolrich et al., 2009).

#### 2.3.2 Volumetric and cortical thickness (CT)

*Volumetric and cortical thickness (CT)* measurements were conducted using FreeSurfer (version 6.0.0) (Dale et al., 1999, Desikan et al., 2006, Livingston et al., 2020). In summary, the preprocessing steps included removing non-brain tissue via surface deformation, segmenting subcortical gray matter structures, correcting for motion artifacts, normalizing intensity, and performing automated Talairach transformation. For subcortical volumes, values for bilateral regions of interest were averaged across the left and right hemispheres.

#### 2.3.3 Fractal dimensionality (FD)

*Fractal dimensionality (FD)* is a scale-invariant mathematical measure that quantifies the complexity of shapes at both macroscopic and microscopic levels, often interpreted as geometric fractals. FD was calculated for various brain regions, including the cortical ribbon (unparcellated gray matter), frontallobe, temporal lobe, occipital lobe, parietal lobe, and subcortical structures, using the calcFD toolbox^4^ (Madan and Kensinger, 2016, Madan and Kensinger, 2017). The calcFD toolbox, compatible with intermediate files from the FreeSurfer pipeline, calculates the FD of 3D brain structures. This is achieved through the box-counting algorithm, where the brain is treated as a 3D structure within a grid. Voxels containing portions of the structure are counted, and the process is repeated iteratively with progressively larger box sizes, with the filled boxes being recounted at each step. The relationship between box sizes and their respective counts are fitted using a power-law model, where the resulting FD is the exponent of the fitted model (Madan and Kensinger, 2016, Madan and Kensinger, 2017).

#### 2.3.4 Diffusion tensor imaging (DTI)

Diffusion weighted images were corrected for eddy current-induced distortion, susceptibility-induced distortion, and motion correction using TOPUP and EDDY tools in FSL (Andersson et al., 2003). DTI metrics, including fractional anisotropy (FA) and mean diffusivity (MD), were calculated using DTIFIT tool in FSL (Behrens et al., 2003). Free water (FW) index and free water corrected DTI metrics (FA and MD) were computed with a bi-tensor model applied to the DWI data, following a previously established algorithm (Dumont et al., 2019). The processing pipeline was implemented using Nextflow pipeline (Di Tommaso et al., 2017) with all software dependencies encapsulated in a Singularity Container (Kurtzer et al., 2017). In this work, free water corrected FA and MD metrics were used.

#### 2.3.5 Cerebral blood flow (CBF) and cerebrovascular reactivity (CVR)

ASL images were processed using the Oxford ASL tool (Chappell et al., 2008). Preprocessing steps included motion correction with MCFLIRT, slice-timing correction, distortion correction, spatial regularization, partial volume correction, and registration to high-resolution anatomical space using the boundary-based registration (BBR) algorithm. Additionally, images were registered to 2 mm MNI-152 standard space using FNIRT nonlinear registration (Greve and Fischl, 2009, Glasser et al., 2022). Cerebral blood flow (CBF) quantification was performed in three steps: (1) Bayesian inference for CBF based on the Buxton kinetic curve model, (2) Bayesian inference of additional parameters applicable to single-post label delay data, and (3) Bayesian inference with spatial priors to refine model parameters, initialized with high-resolution anatomical image (Buxton et al., 1998, Chappell et al., 2008).

CVR maps were calculated using the global signal regression coefficient method applied to rs-fMRI BOLD images (Liu et al., 2017), providing qualitative reactivity indices for each subject at each study visit. Preprocessing steps included motion correction, spatial smoothing with a Gaussian filter [full-width at half-maximum (FWHM) of 4 mm], and linear detrending. Building on previous findings in healthy controls that linked global resting-state BOLD fluctuations to natural variations in ETCO2 levels within the 0.02–0.04 Hz frequency range, we applied a band-pass filter to isolate this specific frequency range in the rs-BOLD data. Gray matter tissue segmentation was used as a mask to compute the global average gray matter rs-fMRI signal, which served as a regressor for voxel-wise BOLD signals. The global signal, previously demonstrated to reflect respiratory and cardiac fluctuations, was regressed against the preprocessed BOLD signal at each voxel. The slope of this regression was taken as the CVR index. Finally, normalization of this index by the whole-brain average provided a qualitative measure of CVR (Liu et al., 2017).

#### 2.3.6 ROI analysis

T1-weighted images were used for co-registration of the DTI, rs-fMRI, and perfusion MR images to anatomical and standard spaces. Tissue maps were used to account for partial volume effects in functional processing. Regions of interests (ROIs) were determined based on previous literature (Tate et al., 2011, Thompson and Jahanshad, 2015, Uddin et al., 2021, Minosse et al., 2023, O’Connor et al., 2023, Uddin et al., 2024)and extracted using the Harvard-Oxford cortical and subcortical atlases available in FSL. Each ROI was warped from standard to native spaces and binarized before being applied as a mask against each imaging metric map. The mean and standard deviation of each metric were calculated using FSL’s *fslstats* tool within each ROI. The following ROIs were included in the analysis. Cortical gray matter: Frontal Pole (FRP), Pars Opercularis (IFG_O), Superior Temporal Gyrus (STG), Postcentral Gyrus (POC), Parahippocampal Gyrus (PHG), Lingual Gyrus (LNG), Insula (ICX), Lateral Occipital Lobe (LOC), Temporal Pole (TMP), Superior Parietal Lobule (SPL), Precuneus (PUC); subcortical gray matter: Caudate Nucleus (CN), Putamen (PUT), Globus Pallidus (GP), Thalamus (TH), Amygdala (Amyg), Accumben Nucleus (AccN), Hippocampus (Hippo); white matter: Splenium of Corpus Callosum (SCC), Genu of Corpus Callosum (GCC), Body of Corpus Callosum (BCC), Posterior Limb of Internal Capsule (PLIC), Anterior Limb of Internal Capsule (ALIC), Superior Longitudinal Fasciculus (SLF), Anterior Corona Radiata (ACR). Bilateral ROIs were merged prior to calculating metric means.

## 3 Statistical methods

We analyzed the following datasets: Cortical gray matter—CT, FD, FW, FA, MD, CBF, CVR; subcortical gray matter—volumes, FD, FW, FA, MD, CBF, CVR; white matter—FW, FA and MD. A total of 147 candidate imaging features, representing four imaging modalities: volumetric measures (volumes, cortical thickness and FD); Diffusion MRI (FW, FA, and MD); perfusion MRI (CBF), and rs-fMRI-based cerebrovascular reactivity (CVR). Out of them, 77 are collected from ROIs in cortical gray matter regions, 49 are from subcortical gray matter regions, and 21 are from white matter regions.

For each feature, we apply an initial screening using a two-sample Welch t-test for identifying mean differences between the PWH group and healthy controls at the baseline visit (*n* = 86 subjects). Given the high-dimensional nature of our study, we adopt a liberal selection threshold of *p* < 0.2. This “soft” screening method is commonly used in high-dimensional statistical learning to retain features with relatively weak marginal associations for model development. A feature is selected for further analysis if the resulting *p*-value is less than 0.2. Based on this criterion, we identified 39 (11 cortical gray matter, 23 subcortical gray matter, and 5 white matter) features to be used for multimodal integrative analysis. A complete list of these features, an ROI-abbreviation legend, and the corresponding results from Welch *t*-test are provided in Supplementary Table 1.

### 3.1 Multimodal integrative analysis

Two outcome variables are considered in this study. The first, denoted as *Z_0_*, is the overall cognitive z-score measured at baseline. This data is available for both PWH and controls (*n* = 86). The second, denoted as Δ*Z*, is the temporal change in z-scores between the baseline and the second visit following 12 weeks of cART treatment. This data is defined only for PWH and is available for 27 subjects (three PWH lacked cognitive data at the 12-week follow-up).

Our main objective was to develop interpretable, regression-based predictors for the two outcome variables. As a secondary objective, we identify the features, as well as the corresponding brain regions and imaging modalities, that are most informative for predicting these outcome variables. Due to the large number of features used in this study, it is reasonable to consider applying dimensionality reduction and model selection steps in training the predictive models for the two outcome variables. Below, we summarize the two approaches we attempted in this study.

- Model 1: We apply the first layer of principal component analyses (PCA) separately on the set of features collected in each brain region (cortical gray matter, subcortical gray matter, and white matter). A second layer of PCA is then applied to the principal components (PCs) defined in the first step to further reduce the dimensionality. For both principal component analyses, numbers of top PCs are selected by the proportion of variance explained (PVE), with the following two options: PVE = 0.8 (80% of total variance) or PVE = 0.9 (90% of total variance). Top PCs defined in the second PCA are used as features in multiple regression models to predict the two outcome variables in the output model. A bi-directional stepwise model selection procedure based on Akaike Information Criterion (AIC) is applied to reduce the complexity and prevent overfitting for the output model.
- Model 2: Much like Model 1, except that top PCs identified in the first layer PCA are used directly as features in the output model to predict the outcome; no second layer PCA is applied.

Of note, we also explored an alternative approach with the output regression model replaced by an elastic-net regression, implemented in R package *glmnet*. However, its performance was significantly worse than our primary approach; therefore, this approach is not presented here.

**FIGURE 1:**
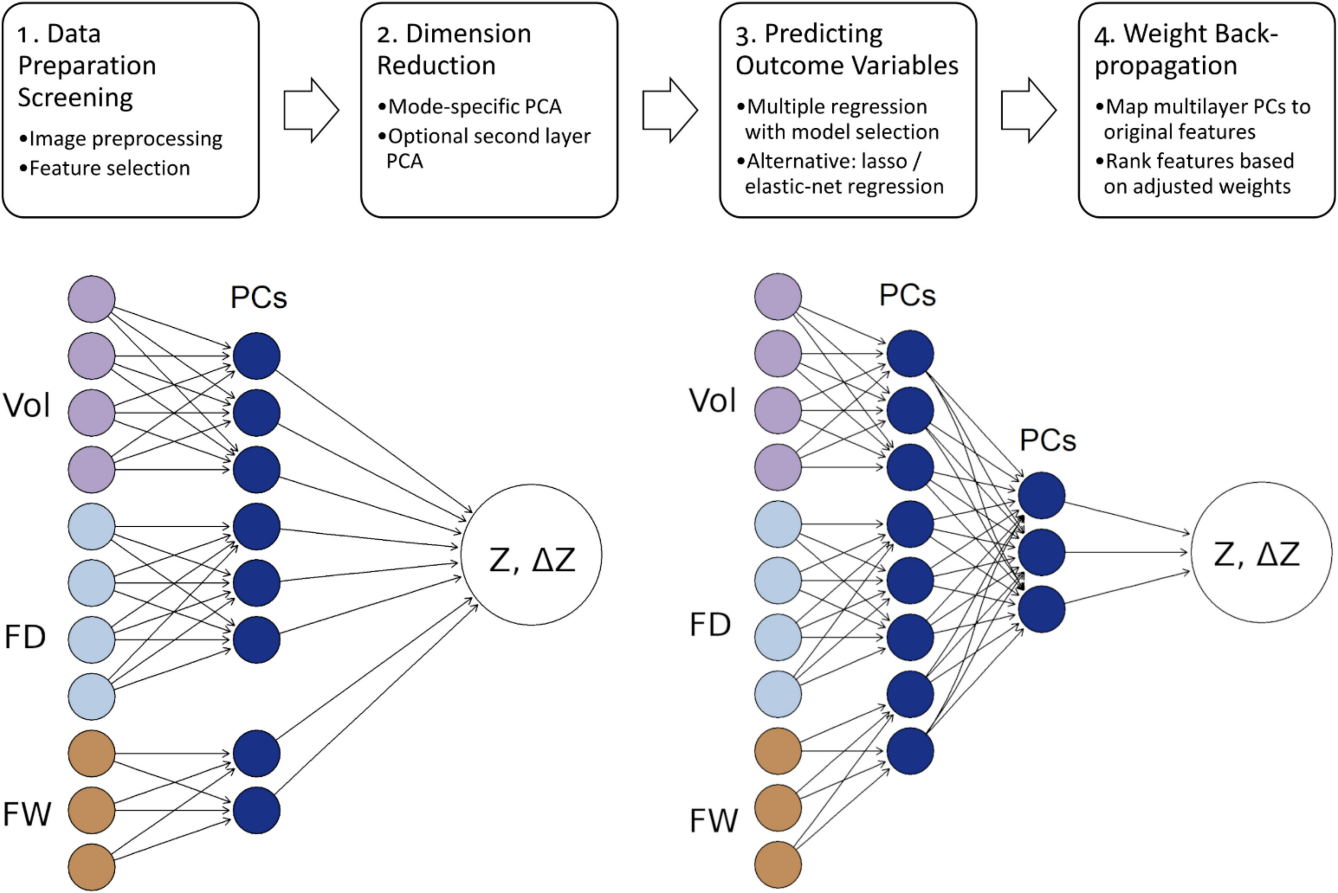
**Top row:** Schematic representation of the analytic pipeline. After image preprocessing and feature selection, principal components (PCs) are extracted separately for each imaging modality. These modality-specific PCs may be concatenated and optionally reduced via a second-layer (global) PCA. Retained PCs (plus any covariates) are used to fit multiple linear regression model (with model selection or penalty) to predict either the baseline cognitive score (*Z*) or 12-week changes (Δ*Z*). Finally, linear coefficients in the PC space are algebraically back-propagated to produce equivalent and scale-adjusted input-space weights (*w_j_*). These adjusted feature weights represent changes in predicted outcome per one standard-deviation change in feature *j*, which can be used to rank features across regions and modalities. **Bottom left:** An illustration of the per-modality PCA →regression pipeline (Vol, FD, FW are example modalities). **Bottom right:** An alternative approach with an additional global PCA step to further reduce modeling complexity.

A schematic representation of this multimodal integrative analysis pipeline is shown in Figure 1.

### 3.2 Unimodality and modality-region models for Δ*Z*

One drawback of the multimodality models is that, by incorporating all imaging modalities across all brain regions, they do not reveal which specific modality or region is most predictive of the outcome variables. To address this deficiency, we developed unimodality models for each of the eight imaging metrics used in this study: Vol, CT, FD, FW, FA, MD, CBF, and CVR. Each model uses only features in that category, selected by the marginal screening procedure based on Welch two sample *t*-test for HIV status. Compared with multimodality models, these unimodality models use far fewer features; therefore, there is no need for dimensionality reduction prior to regression. We decided to employ the same AIC-based stepwise selection procedure to exclude uninformative features and reduce model complexity.

In addition, we developed 17 experimental modality-region models, each using just one modality from a single brain region, e.g., FW measures from the cortical region, to predict *Z* or Δ*Z*. Because each modality-region contains far fewer features than the pooled unimodality models, we decided to skip the feature screening step based on HIV status. As a result, modality-region models reflect the general association between various imaging markers to longitudinal changes of cognitive scores, not just those strongly related to the HIV infection revealed in multimodality and unimodality models. Again, modality-region models use relatively few features; therefore, we do not need to apply a dimensionality reduction procedure before regression analyses. The same AIC-based stepwise selection procedure is used to keep model complexity in check.

#### 3.2.1 Assign weights to original features for multimodality models

Although unimodality and modality-region models offer insight into which specific modalities and brain regions are most associated with cognitive outcomes, these models are certainly not equivalent to the superior multimodality models, because each such model can only use a small subset of features. In this section, we introduce an algorithm that assigns equivalent weights to the original features in multimodality models, allowing us to rank these imaging measures directly.

Mathematically, the final predictor of our multimodality models can be represented as follows:


$${\hat{Y}}_{i}={\hat{\beta }}_{0}+\overset{L}{\underset{l=1}{\sum }}{\text{PC}}_{i\,l}\,{\hat{\beta }}_{l}.$$

Here ${\hat{Y}}_{i}$ is the predicted outcome value (*Z*_0_ or Δ*Z*) for the *i*th subject; PC_*il*_ is the *l*th PC score pertain to the *i*th subject; ${\hat{\beta }}_{0}$ is the intercept, and ${\hat{\beta }}_{l}$ are the linear coefficients associated with PC_⋅*l*_. For Model 1, PC_*il*_ are the second-layer PC scores; for Model 2, PC_*il*_ are scores of the first and only PCA.

By design, ${\hat{\beta }}_{l}$ represents the importance of the *l*th PC, not the original features (denoted as *x*_*ij*_ for the *i*th subject and the *j*th feature). Because PC_⋅*l*_ is a linear combination of all *x*_*⋅j*_, it is not obvious how much each *x*_*ij*_ contributes to the predicted value ${\hat{Y}}_{i}$.

A weight back-propagation algorithm originally developed in our recent study (McCall et al., 2021) enables direct interpretation of our multilayer PC–regression models by mapping principal-component weights ($\hat{\beta_{l}}$) back to the original feature space. Specifically, it computes an equivalent weight vector $\tilde{\beta }=\left({\tilde{\beta }}_{0},{\tilde{\beta }}_{1},\ldots ,{\tilde{\beta }}_{p}\right)$, such that ${\hat{Y}}_{i}={\hat{\beta }}_{0}+\sum_{l=1}^{L}{\text{PC}}_{i\,l}\,{\hat{\beta }}_{l}={\tilde{\beta }}_{0}+\sum_{j=1}^{p}X_{i\,j}\,{\tilde{\beta }}_{j}$, where *X*_*ij*_ is original feature *j* for subject *i*. Due to this mathematical equivalence, the magnitude of $\left|{\tilde{\beta }}_{j}\right|$ quantifies the contribution of *X*_*⋅j*_ to the predictions.

In this study, we introduce two important refinements tailored to multimodality imaging analysis:

- PCA on the Pearson correlation matrix. Because our imaging features span many different modalities with very different natural scales, we apply a variant of PCA based on the eigen-decomposition of the *sample Pearson correlation matrix* instead of sample covariance matrix. This choice prevents any single modality from dominating the principal components, thus ensures a balanced representation of all modalities in top PCs.
- Standard-deviation-adjusted weights. Instead of ranking the importance of image metrics by $\left|{\tilde{\beta }}_{j}\right|$ directly, we define the adjusted weights to be $w_{j}:={\hat{\sigma }}_{j}{\tilde{\beta }}_{j}$, where ${\hat{\sigma }}_{j}$ is the sample standard deviation of *X*_*⋅j*_. By construction, *w_j_* represents the expected change in the predicted value (${\hat{Y}}_{i}$) associated with one STD change in *X*_*⋅j*_, which remains invariant under any rescaling of *X*_*⋅j*_. This invariance makes these adjusted weights directly comparable across features and modalities.

Based on these refinements, the adjusted weights are more appropriate than the unadjusted weights for prioritizing features with vastly different scales. In Tables 2, 3, we see that the adjusted weights are much more comparable to each other than the unadjusted weights.

**TABLE 2:** Equivalent weights of imaging features in Model 2 (with PVE = 0.9) for predicting the baseline total Z-scores (*Z*) using baseline data from *n* = 86 subjects.

Features	Adjusted weights (*w*_*j*_)	Unadjusted weights (${\tilde{\beta }}_{j}$)	Mean	STD
Amyg.CBF.sub	–0.864	–6.632	0.828	0.130
PUC.CVR.cor	0.742	1.873	1.579	0.396
GP.Vol.sub	–0.718	–45.122	0.131	0.016
PUT.FW.sub	0.699	132.502	0.008	0.005
CN.MD.sub	0.568	13407.930	0.001	0.000
GP.FA.sub	–0.541	–22.311	0.372	0.024
AccN.CBF.sub	0.501	2.394	1.261	0.209
CN.Vol.sub	0.501	15.797	0.241	0.032

Features reported in this table are ranked by the adjusted weights (w_j_) and selected based on |*w*_*j*_| = 0.5. Features are represented as ROI.MRI-metric.region. ROIs: Amyg–Amygdala; PUC, Precuneus; GP, Globus Pallidus; PUT, Putamen; CN, Caudate Nucleus; AccN, Accumben Nucleus. Metrics: CBF, cerebral blood flow; CVR, cerebrovascular reactivity; Vol, subcortical volume; FW, extracellular free water; MD, mean diffusivity; FA, fractional anisotropy. Regions: sub, subcortical gray matter; cor, cortical gray matter.

**TABLE 3:** Equivalent weights of imaging features in Model 2 (with PVE = 0.9) for predicting the longitudinal changes of total Z-scores (Δ*Z*) using longitudinal data from n = 27 subjects.

Features	Adjusted weights (*w*_*j*_)	Unadjusted weights (${\tilde{\beta }}_{j}$)	Mean	STD
ALIC.FW.wm	4.338	901.143	0.002	0.005
LNG.FD.cor	–1.798	–53.268	2.089	0.034
Hippo.CVR.sub	1.704	6.169	0.978	0.276
PUT.FW.sub	–1.494	–283.028	0.008	0.005
ICX.CBF.cor	–1.284	–8.208	1.259	0.156
Hippo.FW.sub	–1.211	–25.513	0.208	0.047
Hippo.FA.sub	–1.058	–57.328	0.223	0.018
TH.CVR.sub	–0.960	–2.759	0.922	0.348
GP.Vol.sub	0.953	59.870	0.131	0.016
CN.Vol.sub	–0.916	–28.895	0.241	0.032
IFG-O.CBF.cor	–0.867	–4.016	1.589	0.216
AccN.FA.sub	–0.850	–56.733	0.184	0.015
Amyg.MD.sub	0.818	4299.261	0.001	0.000
IFG-O.CT.cor	–0.816	–5.567	2.640	0.147
FRP.FA.cor	–0.803	–55.027	0.129	0.015
FRP.CBF.cor	0.735	2.866	1.245	0.257
Amyg.FW.sub	–0.714	–19.008	0.172	0.038
Amyg.CBF.sub	0.701	5.378	0.828	0.130
AccN.CBF.sub	–0.700	–3.344	1.261	0.209
PLIC.FA.wm	0.661	28.748	0.578	0.023
PUT.CVR.sub	0.603	1.916	0.811	0.315
PUT.CBF.sub	0.595	4.400	1.003	0.135

Features reported in this table are ranked by the adjusted weights (w_j_), and selected based on |*w*_*j*_| = 0.5. Features are represented as ROI.MRI-metric.region. ROIs, ALIC, anterior limb of internal capsule; LNG, lingual gyrus; Hippo, hippocampus; TH, thalamus; GP, Globus Pallidus; CN, Caudate Nucleus; IFG-O, pars opercularis; AccN, Accumben Nucleus; Amyg, Amygdala; FRP, frontal pole; PLIC, posterior limb of internal capsule; PUT, Putamen. Metrics: FD, fractal dimensionality; CVR, cerebrovascular reactivity; FW, free water; CBF, cerebral blood flow; FA, fractional anisotropy; Vol, volume; MD, mean diffusivity; CT, cortical thickness. Regions: wm, white matter; cor, cortical gray matter; sub, subcortical gray matter.

#### 3.2.2 Model evaluation

Because our sample was small, we did not perform cross-validation. Instead, we evaluate and rank fitted models using adjusted *R*^2^, which corrects the ordinary (in-sample) *R*^2^ for model complexity and is therefore a fair quantity for comparing models that differ in the number of predictors.

Let *n* be the sample size, *p* be the number of predictors (excluding the intercept), $\text{SST}=\sum_{i=1}^{n}{\left(Y_{i}-\bar{Y}\right)}^{2}$ be the total sum of squares, $\text{SSE}=\sum_{i=1}^{n}{\left(Y_{i}-\hat{Y_{i}}\right)}^{2}$ be the error sum of squares, the adjusted *R*^2^ is defined as


$$\text{Adjusetd}\,R^{2}=1-\frac{\text{SSE}/\left(n-p-1\right)}{\text{SST}/\left(n-1\right)}.$$

For readers who prefer an error-scale measure, we also report the residual standard error (RSE), defined as $\text{RSE}=\sqrt{\text{SSE}/\left(n-p-1\right)}$, in Supplementary Text for each fitted model. Since we are comparing models’ fit to the same outcome variable, SST/(*n* − 1) is the same across models. Therefore, adjusted *R*^2^ is a monotonic decreasing function of RSE, so these two metrics of model fitting lead to the same ranking of models. In contrast, unadjusted model fitting measures such as SSE or mean squared error (MSE = SSE/*n*) do not account for different degrees of freedom thus tend to favor more complex models. Therefore, they are not appropriate for the model comparisons presented here.

## 4 Results

### 4.1 Multimodality models

#### 4.1.1 Modeling baseline z-scores with multimodality models

We apply both Model 1 and Model 2 (using PVE = 0.8 and 0.9) to assess the relationship between imaging features and baseline total Z-scores (*Z*). Model 2 provides the best fit, with an adjusted *R*^2^ = 0.0952 (using PVE = 0.8) and 0.145 (using PVE = 0.9), both are higher than Model 1’s adjusted *R*^2^ values of 0.0411 (PVE = 0.8) and 0.0749 (PVE = 0.9).

**FIGURE 2:**
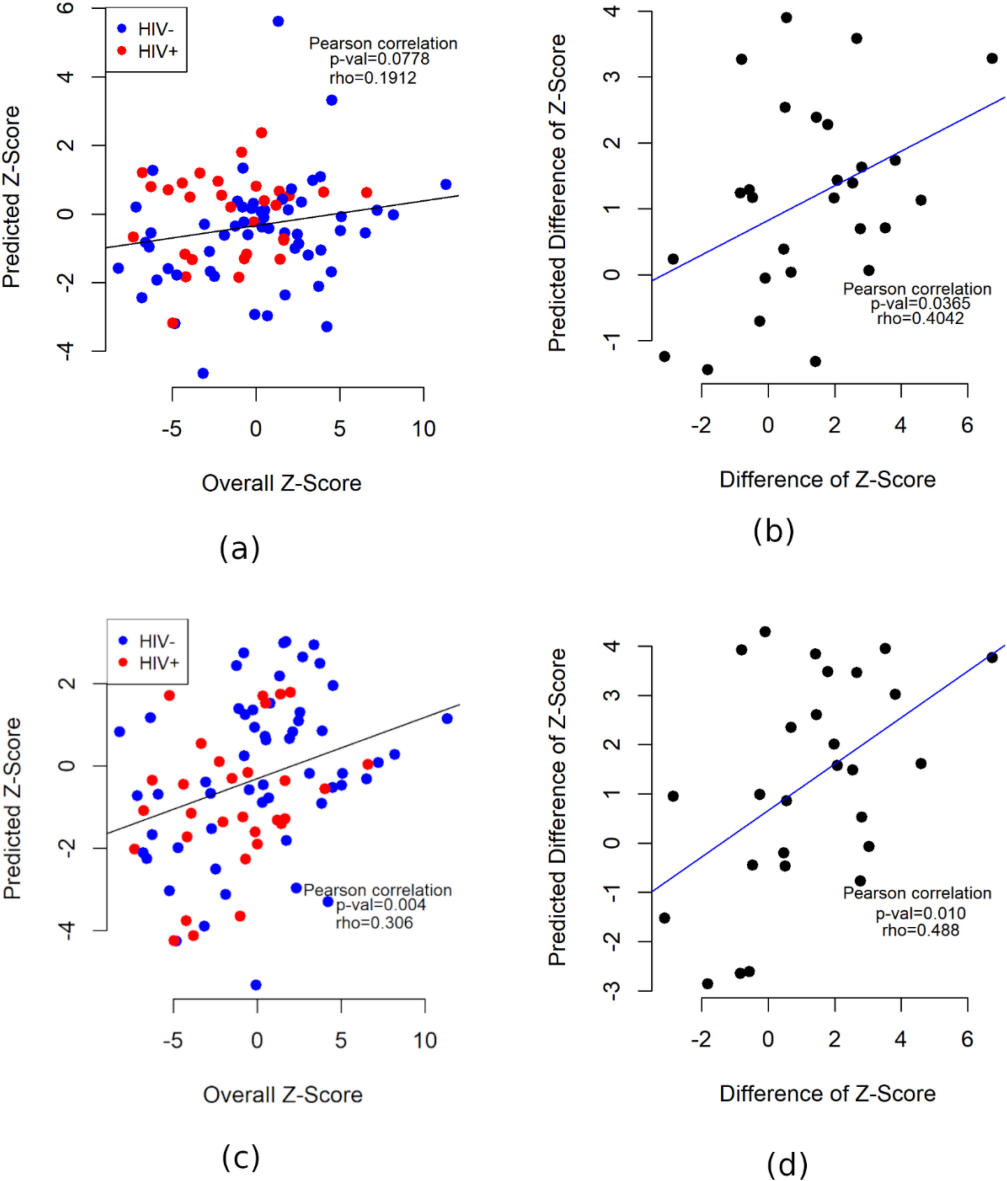
Scatter plots showing the association between the observed and predicted cognitive outcome values (**Z** and Δ**Z**). For both Models 1 and 2, PVE = 0.9 is used. **(a)** Model 1 for **Z**, *n* = 86. **(b)** Model 1 for Δ**Z**, *n* = 27. **(c)** Model 2 for **Z**, *n* = 86. **(d)** Model 2 for Δ**Z**, *n* = 27.

The Pearson correlation between the observed Z-scores and those fitted by Model 2 (PVE = 0.9) is ρ = 0.306 (*p* = 0.004), suggesting a modest overall association between various imaging features and the baseline cognitive performance. Scatter plots of observed *Z* versus predicted values from both Model 1 and 2 are presented in Figures 2a,c.

We apply the equivalent weight back-propagation algorithm to Model 2 (PVE = 0.9). Most top features (ranked by the adjusted weights) are derived from subcortical ROIs, e.g., Amyg, GP, and PUT, as shown in Table 2. The clinical significance of this finding aligns well with the known pathology of HIV-associated neurocognitive impairment targeting subcortical gray matter (Nir et al., 2021, McMahan et al., 2023).

Notably, the unadjusted weights exhibited significant variability across features, making them less reliable for ranking purposes. In contrast, the adjusted weights defined in this study proved to be numerically stable and thus more suitable for identifying the most predictive features.

Detailed results of these approaches, including number of PCs selected in the last step predictive model and their goodness-of-fit metrics, are provided in Supplementary Text.

#### 4.1.2 Modeling longitudinal changes of z-scores with multimodality models

We apply both Model 1 and Model 2 (using PVE = 0.8 and 0.9) to assess the relationship between imaging features and longitudinal changes of total Z-scores (Δ*Z*). Similar to modeling *Z*, we find that the best fit is provided by Model 2 using PVE = 0.9, which attains an adjusted *R*^2^ = 0.6339 for predicting Δ*Z*.

The Pearson correlation between the observed Δ*Z* and those fitted by Model 2 (PVE = 0.9) is ρ = 0.488 (*p* = 0.01), which is much higher than the corresponding association between imaging features and *Z*, also produced by Model 2. Scatter plots of observed Δ*Z* versus predicted values from both Model 1 and 2 are presented in Figures 2b,d.

We apply the equivalent weight assigning algorithm to Model 2 (PVE = 0.9), and the results are shown in Table 3. Compared with Table 2 (modeling baseline Z-scores), we noticed that: (a) Many more features are deemed important (based on |*w*_*j*_| = 0.5) for modeling Δ*Z*, and (b) while subcortical ROIs such as Amyg and PUT are also key players in Table 3, many features from cortical gray matter and white matter regions are presented in this list. This transition from a subcortical-dominant effect at the baseline to a more widely spread multimodal effect suggests that while initial cognitive impairment is largely driven by subcortical regions, the trajectory of recovery after 12-weeks cART treatment is a much broader, systematic process.

Detailed results of these analyses are provided in Supplementary Text.

#### 4.1.3 Results of unimodality models for Δ*Z*

Among all unimodality models (CT, FD, Vol, FW, FA, MD, CBF, and CVR), the CBF model achieves the best fit with an adjusted *R*^2^ = 0.3118, which is still significantly less than the adjusted *R*^2^ = 0.6339 of the multimodality model. Out of six candidate features used in this model (FRP, IFG_O, and ICX in the cortical region; PUT, Amyg, and AccN in the subcortical region), two are deemed informative by the model selection procedure: PUT ($\hat{\beta }=12.562$, *p* = 0.0019) and Amyg ($\hat{\beta }=-12.4607$, *p* = 0.004).

The second best unimodality model is FA, with an adjusted *R*^2^ = 0.2162. Out of eight candidate features used in this model (FRP in the cortical region; GP, TH, Amyg, AccN, and Hippo in the subcortical region; PLIC and ACR in the white matter), five are kept by the model selection procedure: FRP ($\hat{\beta }=-60.4569$, *p* = 0.2027), Amyg ($\hat{\beta }=100.467$, *p* = 0.0458), AccN ($\hat{\beta }=-68.8955$, *p* = 0.0269), Hippo ($\hat{\beta }=-64.087$, *p* = 0.1571), and PLIC ($\hat{\beta }=-54.6887$, *p* = 0.0128). It is worth noting that AIC-based model selection prioritizes the overall information content of the model rather than the independent significance of a single variable. Retained features with insignificant *p*-value (e.g., FRP and Hippo) may provide complementary information that, when combined with other more significant predictors, improves the model’s overall predictability and generalizability.

The 3rd, 4th, and 5th best models are FW (adjusted *R*^2^ = 0.1972; selected features are Amyg and Hippo), CVR (adjusted *R*^2^ = 0.1971; selected features are PUT and TH), MD (adjusted *R*^2^ = 0.1176, selected features are FRP, ICX, and PLIC). No informative features are selected in Vol, CT and FD models.

Based on these results, we conclude that:

1. No single imaging modality shows a stronger association with Δ*Z* (cART-induced changes in cognitive performance) than the multimodality model. It suggests that cognitive recovery after cART treatment is a systematic process that requires the integration of diverse brain signals.
2. CBF and FA are more strongly associated with Δ*Z* than other modalities (CT, FD, FW, CVR, MD, and Vol). This likely reflects early hemodynamics and microstructural changes associated with HIV infection.
3. Amyg (subcortical) appears to be the most informative ROI, since it is selected by three unimodality models (CBF, FA, FW). This high consistency suggests that the Amygdala may be an important “hub” for the response to cART treatment.
4. Other ROIs selected by more than one model, suggesting diffuse involvement, include: PUT (subcortical, selected by CBF and CVR); FRP (cortical, FA and MD), Hippo (subcortical, FA and FW), PLIC (white matter, FA and MD).

Detailed results of these models, including selected informative ROIs, estimated linear coefficients and their *p*-values, unadjusted *R*^2^, and the residual sum of squares (RSS), are provided in Supplementary Table 2.

#### 4.1.4 Results of modality-region models for Δ*Z*

We developed 17 modality–region models, using the following feature sets for each region type:

1. Cortical region: CT, FD, FW, FA, MD, CBF, and CVR.
2. Subcortical region: Vol, FD, FW, FA, MD, CBF, and CVR.
3. White matter: FW, FA, and MD.

Among them, the top four models (ranked by adjusted *R*^2^) are all based on cortical features:

1. FD/cortical (adjusted *R*^2^ = 0.4753; selected ROIs: FRP, ICX, LOC, SPL)
2. CVR/cortical (adjusted *R*^2^ = 0.4065; FRP, POC, STG, PHG)
3. CT/cortical (adjusted *R*^2^ = 0.3774; IFG_O, LOC)
4. CBF/cortical (adjusted *R*^2^ = 0.3767; ICX, STG)

The 5th and 6th best models use subcortical features:

1. FW/subcortical (adjusted *R*^2^ = 0.3241; CN, TH, Amyg, Hippo)
2. CBF/subcortical (adjusted *R*^2^ = 0.3118; PUT, Amyg). Of note, the CBF/subcortical model is identical to the best unimodality model, the CBF model.

Models that use white matter features do not perform well. The best such model is MD/white matter, which has an adjusted *R*^2^ = 0.1325. FW/white matter and FA/white matter have adjusted *R*^2^ of 0.1089 and 0.00438, respectively. In addition, no informative features are selected for four models: FA/cortical, Vol/subcortical, FD/subcortical, and MD/subcortical.

Based on these results, we conclude that:

1. No modality-region model shows a stronger association with Δ*Z* (cART induced changes in cognitive performance) than the multimodality model. It confirms that while there are localized features correlated with Δ*Z*, the most robust prediction of cognitive improvement is made by integrating diverse signals across the entire brain.
2. Four modality-region models achieve better association with Δ*Z* than the best unimodality model in terms of the adjusted *R*^2^. This is likely because the feature screening based on HIV status was not applied to modality-region models, allowing them to utilize features not associative with HIV status.
3. All four top-performing modality-region models use cortical features (e.g., FRP, ICX, LOC, and STG), whereas subcortical ROIs (e.g., Amyg, PUT, and Hippo) play a more important role in unimodality models. These subcortical regions are central to the frontostriatal and limbic circuits that are particularly susceptible to HIV-related inflammation and injury. Our results suggest that while HIV-related injury is preferentially localized in subcortical structures, the longitudinal trajectory of cognitive recovery is more dependent on cortical gray matter integrity, as measured by FD and cerebral vascular reactivity (CVR).

Detailed results of these models are provided in Supplementary Table 3.

## 5 Discussion

Cognitive impairment is a common finding in HIV infection and has persisted despite the use of Cart (Heaton et al., 2010). Improvement and worsening in cognitive performance have been reported in longitudinal studies (Heaton et al., 2015). The biggest change in cognitive performance is expected to occur in those PWH that are cART naïve starting treatment or failing a cART regimen and starting a new one. The assumption is that HIV-associated chronic inflammation is minimized by cART, thus reducing the risk of brain injury (Gisslén et al., 2016, Rahimy et al., 2017). Accordingly, the cohort of this study included cART naïve participants followed longitudinally after initiating treatment (Weber et al., 2022). Although the availability of multimodality imaging within an acceptable scanning time makes it possible to integrate multiple imaging data, the benefit of such an approach remains to be demonstrated.

The imaging modalities metrics used in the analyses, morphometry (Nir et al., 2019, Weber et al., 2022), DTI (Zhu et al., 2013, Ragin et al., 2015), CBF (Ances et al., 2009, Su et al., 2017, Singh et al., 2023), CVR (by several approaches(Chow et al., 2016, Callen et al., 2020, Singh et al., 2023), and extracellular free water (Uddin et al., 2021), have been previously utilized to measure brain injury in PWH.

Our study demonstrates the utility of multimodal integrative models for associating diverse brain imaging metrics with neurocognitive outcomes in PWH undergoing cART treatment. By combining advanced statistical approaches with multimodal imaging data, we provide important insights into the predictive value of MRI metrics for baseline cognitive performance and longitudinal changes during treatment.

Because our dataset is “large p, small n,” it is critically important to employ robust and conservative statistical methods to control model complexity and prevent model overfitting. This is why we incorporated a three-step integrative analysis consisting of marginal feature screening, multi-layered PCA for dimensionality reduction, and AIC-based model selection. To evaluate these models, we report Adjusted *R*^2^ rather than the residual sum of squares (RSS), because the adjusted *R*^2^ provides a mathematically explicit correction for model complexity (*p*) relative to sample size (*n*), which makes it a fair quantity for comparing models with different degrees of freedoms. Furthermore, we developed a novel equivalent weight back-propagation method to “translate” a complex multi-step predictive model into an equivalent regression model using the original features and rank them based on the adjusted weights that quantify their contributions to the predictive model.

Using this multi-step statistical approach, we identified a modest association (adjusted *R*^2^ = 0.145; 95% CI: [-0.084, 0.255]) between various imaging metrics and baseline neurocognitive scores (*Z*). The weight back-propagation analysis shows that subcortical ROIs, such as Amyg and PUT, play a dominant role in explaining the variation of baseline cognitive scores.

In comparison, we observed a much stronger association [adjusted *R*^2^ = 0.6339; 95% CI: (0.170, 0.835)] between imaging markers and Δ*Z* (longitudinal changes of cognitive scores following 12 weeks of cART treatment for PWH). Notably, imaging features from cortical gray matter and white matter regions are also identified as informative in the equivalent weight analysis for Δ*Z*. To get an in-depth understanding of this strong association between imaging features and Δ*Z*, we further developed unimodality and modality-region models to predict Δ*Z*. The results of these models are broadly consistent with the multimodality model. However, the multimodality model provided superior predictive performance, as indicated by its higher adjusted *R*^2^ values and better model fit. This finding underscores the importance of integrating diverse imaging features, which likely reflect distinct but interrelated aspects of brain pathology in PWH. Further, this approach maximizes the ability to examine large data sets with small sample sizes. For instance, a power analysis for a multivariate regression with ten covariates using Cohen’s *f*^2^ = 0.906 derived from the best modality-region model (FD/cortical, adjusted *R*^2^ = 0.4753), reveals that *n* = 33 subjects would be needed to achieve 90% power at the significance level α = 0.05. In contrast, with *f*^2^ = 1.731 derived from the multimodal model (adjusted *R*^2^ = 0.6339), only *n* = 23 subjects are needed for the same statistical power. The multimodal imaging approach reduces the required sample size by roughly 30%, showing the clear advantage for studies with small cohorts.

The strong performance of multimodality models emphasizes the importance of collecting diverse imaging modalities to better understand and predict neurocognitive trajectories in PWH. In this regard, we have observed differences in PWH at baseline vs. longitudinal response to cART treatment. The identification of specific ROIs, such as Amyg, PUT (subcortical gray matter) and FRP, LOC (cortical gray matter), suggests potential biomarkers for monitoring cognitive recovery under cART or other interventions aimed at improving cognition. The equivalent weight back-propagation method enhances the interpretability of PCA-based multimodality models by quantifying feature importance. We believe this method could be adapted to other types of data to gain biological insights from complex statistical and machine-learning models in the future.

While multimodal models achieved the best predictive results, unimodality models (Supplementary Table 2) emphasize marginal associations between specific types of features and cognitive outcome. Consistent with multimodal models, those unimodality models based on subcortical perfusion (CBF) and white matter microstructure (FA) yield the best results. These findings suggest that the strongest direct cognitive impact of HIV is localized in subcortical structures. On the other hand, the results of our modality-region models (Supplementary Table 3) showed that cortical gray matter features, such as fractal dimensionality (FD) and cerebrovascular reactivity (CVR), achieved the highest predictive power (adjusted *R*^2^ 0.4753 and 0.4065, respectively). Recall that modality-region models did not face “large p, small n” problem, therefore we skipped the initial HIV-status feature screening, which allowed these models to retain features that were not impaired at baseline but were predictive of longitudinal recovery. This indicates that while cortical regions may not show significant baseline injury, their microstructural complexity (FD) and hemodynamic reactivity (CVR) may still be informative indicators of a patient’s potential for cognitive gain under cART.

Of note, no imaging metrics from certain modalities such as subcortical volume (Vol), cortical thickness (CT), and fractal dimensionality (FD) were selected by the AIC-based procedure for predicting Δ*Z*. While HIV infection is associated with volume loss and cortical thinning in frontostriatal and limbic regions due to chronic inflammation, these *macro-structural* changes are “long-term indicators” that would take a much longer period to be revealed. In comparison, modalities like CBF and FA are more sensitive to fast physiological changes in an initial cART treatment.

Despite these promising findings, our study is not without limitations. First, the sample size, particularly for longitudinal analyses, was relatively small, which may limit the generalizability of our results. Therefore, our work must be considered as exploratory and hypothesis-generating rather than confirmatory. Future studies with larger cohorts and longer follow-ups are needed to validate these findings and explore additional imaging modalities. It is worth noting that the reduction from 137 enrolled participants to 86 subjects used in the multimodality analysis has resulted from the requirement of complete data across all nine domains (eight imaging modalities plus the cognitive assessment). Mathematically, even a modest 5% attrition rate per modality leads to a sizable [1-(1-5%)^9^ ≈ 37%] cumulative subject loss. While future studies might use generative AI or advanced data imputation methods to mitigate this issue, we focus on demonstrating the utility of multimodal data integration based on compete observations in this study.

Second, the PWH cohort was on average younger than the HIV-uninfected controls, which is a difference that emerged from recruitment constraints of the parent study. However, our primary analyses focused on predicting cognitive performance and its short-term change (Δ*Z*) within PWH rather than testing cross-sectional group differences. Consequently, this age imbalance is unlikely to affect our main conclusions.

Third, while cART reduces neuroinflammation, vascular and metabolic factors may significantly moderate the observed cognitive improvements and imaging markers such as CBF and diffusion metrics. However, due to the “large p, small n” nature of our study, incorporating additional clinical factors and their interactions with imaging markers would put even more burden on the control of model complexity. An in-depth study of the intricate interactions between clinical factors and multimodal imaging markers is highly informative but would require a significantly larger sample size to achieve the necessary statistical power.

Lastly, while our multimodality models integrate diverse features, their interpretability relies on statistical methods such as PCA, which can obscure underlying biological mechanisms. The equivalent weight back-propagation method can alleviate this problem, but this method critically depends on the linearity of the dimensionality reduction (PCA) and output model (linear regression). Lastly, we did not compare our pipeline directly with deep-learning approaches. This was deliberate: with our small sample, those complex nonlinear models are prone to overfitting and require extensive tuning to yield reliable out-of-sample performance, and their internal representations are impossible to translate into *exact* per-feature weights. By contrast, the weight back-projection used here yields mathematically equivalent, scale-adjusted feature weights in the original feature space, facilitating biological interpretation needed for region- and modality-level hypothesis generation. A future study with a substantially larger sample would provide sufficient training data to evaluate nonlinear methods with careful regularization, external validation, and interpretability tools, which would allow predictive gains to be weighed against biological insight.

In conclusion, our study demonstrates the potential of multimodality models in predicting neurocognitive outcomes and identifying key imaging biomarkers in PWH undergoing cART. These findings contribute to the growing body of evidence supporting the role of advanced neuroimaging techniques in neuro-HIV research and provide a foundation for future work aimed at improving cognitive health in this population.

## Data Availability

The raw data supporting the conclusions of this article will be made available by the author, without undue reservation.

## Footnotes

1
https://fsl.fmrib.ox.ac.uk/fsl/fslwiki/

2
http://stnava.github.io/ANTs002F

3
https://surfer.nmr.mgh.harvard.edu

4
https://github.com/cMadan/calcFD
