# Supplementary material for: A multimodal imaging–based integrative framework for HIV-associated cognitive impairment and treatment response
Source: Front Neurosci. 2026 Apr 13;20:1775047. doi: 10.3389/fnins.2026.1775047 (PMC13111216; doi:10.3389/fnins.2026.1775047)
Supplement: Supplementary file 1 [file Data_Sheet_1.pdf]

Supplementary Text for “A Multimodal Imaging–Based Integrative Framework for HIV-Associated Cognitive Impairment and Treatment Response”

Model 1

In this approach, we apply the first layer principal component analyses (PCA) separately on the set of features collected in each brain region (cortical gray matter, subcortical gray matter, and white matter). A second layer PCA is then applied to the principal components (PCs) defined in the first step to further reduce the dimensionality. For both principal component analyses, numbers of top PCs are selected by the proportion of variance explained (PVE) with two options: PVE=0.8 (80% of total variance) or PVE=0.9 (90% of total variance). Top PCs defined in the second PCA are used as features in multiple regression models to predict the two outcome variables in the output model. A bi-directional stepwise model selection procedure based on Akaike Information Criterion (AIC) is applied to reduce the complexity and prevent overfitting for the output model.

The following table summarizes number of PCs produced by the 1st and 2nd layer PCA, and number of informative 2nd layer PCs selected by the stepwise model selection procedure for predicting Z (baseline z-scores) and ΔZ (longitudinal changes of z-scores).

	PVE cutoff =0.8			
Brain region (# of features)	# of PCs 1st layer	# of PCs 2nd layer	PCs selected for predicting Z*	PCs selected for predicting ΔZ**
cortical (11)	5	9	PC6	PC1, PC2, PC3, PC4
subcortical (23)	8			
white matter (5)	3			
	PVE cutoff =0.9			
cortical (11)	7	16	PC1, PC4, PC8, PC15	PC1, PC4, PC5, PC8, PC15
subcortical (23)	12			
white matter (5)	4			

* Overall z-score at baseline (n=86)

** Difference of overall z-score between baseline and 12 weeks (n=27)

Below we show the results of regression analysis of Z (**overall z-score at baseline, n=86**) as a function of selected PCs and HIV cohort, using **PVE cutoff=0.8**.

	Estimate	Std.Error	t value	Pr(>|t|)
(Intercept)	0.06704	0.52375	0.128	0.898
cohortHIV+	-1.34862	0.89377	-1.509	0.135
PC6	0.62757	0.39737	1.579	0.118

Residual standard error: 3.903 on 83 degrees of freedom

Multiple R-squared: 0.06367. Adjusted R-squared: 0.04111

F-statistic: 2.822 on 2 and 83 DF, p-value: 0.0652. AIC: 483.2107

Results of regression analysis of Z (**overall z-score at baseline, n=86**) as a function of selected PCs and HIV cohort, using **PVE cutoff=0.9**.

	Estimate	Std. Error	t value	Pr(>|t|)
(Intercept)	-0.4034	0.4133	-0.976	0.3320
PC1	0.4029	0.2669	1.51	0.1350
PC4	0.6439	0.3196	2.015	0.0473
PC8	0.5261	0.379	1.388	0.1688
PC15	0.8078	0.499	1.619	0.1094

Residual standard error: 3.833 on 81 degrees of freedom

Multiple R-squared: 0.1185. Adjusted R-squared: **0.07494**

F-statistic: 2.722 on 4 and 81 DF, p-value: 0.0351. AIC: 482.0238

Results of regression analysis of ΔZ (**longitudinal changes of z-score, n=27**) as a function of selected PCs, using **PVE cutoff=0.8**.

	Estimate	Std. Error	t value	Pr(>|t|)
(Intercept)	0.04157	0.58248	0.071	0.9437
PC1	0.68785	0.35488	1.938	0.0655
PC2	-0.51031	0.27775	-1.837	0.0797
PC3	0.54429	0.26029	2.091	0.0483
PC4	-1.06941	0.4102	-2.607	0.0161

Residual standard error: 1.949 on 22 degrees of freedom

Multiple R-squared: 0.3681. Adjusted R-squared: 0.2532

F-statistic: 3.204 on 4 and 22 DF, p-value: 0.03237. AIC: 119.1258

Results of regression analysis of ΔZ (**longitudinal changes of z-score, n=27**) as a function of selected PCs, using **PVE cutoff=0.9**.

	Estimate	Std. Error	t value	Pr(>|t|)
(Intercept)	1.0092	0.5613	1.798	0.08656
PC1	0.8835	0.3502	2.523	0.01978
PC4	-2.1615	0.535	-4.04	0.00059
PC5	-0.7531	0.3617	-2.082	0.04976
PC8	0.5038	0.3027	1.664	0.11095
PC15	-1.6094	0.6219	-2.588	0.01716

Residual standard error: 1.811 on 21 degrees of freedom

Multiple R-squared: 0.4794. Adjusted R-squared: **0.3555**

F-statistic: 3.868 on 5 and 21 DF, p-value: 0.01217. AIC: 115.8944

For Model 1, using PVE=0.9 yields better performance than PVE= 0.8, as judged by the adjusted R-squared (R^2), which is a measure of model fit that accounts for model complexity. Specifically, with PVE=0.9, Model 1 achieves an adjusted $R^2 = 0.07494$ (p=0.0351) for predicting Z, and 0.3555 (p=0.01217) for predicting ΔZ. Models using PVE=0.8 also achieve better (smaller) AICs than the comparable models using PVE=0.9.

Model 2

In this approach, we apply one PCA to the combined set of all features, then use the top PCs as features in the output model to predict the outcome. **No second layer PCA is applied**. Like Model, two options of PVE cutoffs are attempted: PVE=0.8 (80% of total variance) or PVE=0.9 (90% of total variance).

The following table summarizes number of PCs produced by the first layer PCA and number of informative PCs selected by the stepwise model selection procedure for predicting Z (baseline z-scores) and ΔZ (longitudinal changes of z-scores).

<table>
<tr><td rowspan="2">Brain region (# of features)</td><td colspan="3">PVE cutoff =0.8</td></tr>
<tr><td># of PCs 1st layer</td><td>PCs selected for predicting $Z*$</td><td>PCs selected for predicting $\Delta Z**$</td></tr>
<tr><td>cortical (11)</td><td>5</td><td rowspan="3">sub.PC1, sub.PC2, sub.PC6</td><td rowspan="3">cor.PC4, sub.PC1, sub.PC2, sub.PC5, sub.PC6, wm.PC1, wm.PC3</td></tr>
<tr><td>subcortical (23)</td><td>8</td></tr>
<tr><td>white matter (5)</td><td>3</td></tr>
<tr><td rowspan="2"></td><td colspan="3">PVE cutoff = 0.9</td></tr>
<tr><td># of PCs 1st layer</td><td>PCs selected for predicting $Z*$</td><td>PCs selected for predicting $\Delta Z**$</td></tr>
<tr><td>cortical (11)</td><td>7</td><td rowspan="3">cor.PC5, sub.PC1, sub.PC2, sub.PC5, sub.PC6, sub.PC9, sub.PC10, sub.PC12</td><td rowspan="3">cor.PC1, cor.PC3, cor.PC4, cor.PC7, sub.PC1, sub.PC2, sub.PC4, sub.PC5, sub.PC7, sub.PC8, sub.PC10, sub.PC11, sub.PC12, wm.PC1, wm.PC2, wm.PC3</td></tr>
<tr><td>subcortical (23)</td><td>12</td></tr>
<tr><td>white matter (5)</td><td>4</td></tr>
</table>

* Overall z-score at baseline (n=86)

** Difference of overall z-score between baseline and 12 weeks (n=27)

Results of regression analysis of Z (**overall z-score at baseline, n=86**) as a function of selected PCs and HIV cohort, using **PVE cutoff=0.8**.

	Estimate	Std.Error	t value	Pr(>|t|)
(Intercept)	0.1642	0.5581	0.294	0.7694
cohortHIV+	-1.6271	1.0891	-1.494	0.1391
sub.PC1	0.3141	0.1647	1.907	0.0600
sub.PC2	-0.1535	0.2999	-0.512	0.6102
sub.PC6	0.8866	0.392	2.262	0.0264

Residual standard error: 3.791 on 81 degrees of freedom

Multiple R-squared: 0.1378. Adjusted R-squared: 0.09523

F-statistic: 3.237 on 4 and 81 DF, p-value: 0.01623. AIC: 480.1167

Results of regression analysis of Z (**overall z-score at baseline, n=86**) as a function of selected PCs and HIV cohort, using **PVE cutoff=0.9**.

	Estimate	Std.Error	t value	Pr(>|t|)
(Intercept)	-0.4034	0.3974	-1.015	0.3132
cor.PC5	0.9317	0.4619	2.017	0.0472
sub.PC1	0.2208	0.1514	1.459	0.1486
sub.PC2	-0.4552	0.2403	-1.894	0.0619
sub.PC5	-0.5219	0.3592	-1.453	0.1503
sub.PC6	0.758	0.3896	1.946	0.0553
sub.PC9	0.8295	0.4557	1.82	0.0726
sub.PC10	0.8409	0.5407	1.555	0.1240
sub.PC12	-1.0842	0.6106	-1.775	0.0798

Residual standard error: 3.685 on 77 degrees of freedom

Multiple R-squared: 0.2255. Adjusted R-squared: **0.145**

F-statistic: 2.802 on 8 and 77 DF, p-value: 0.008871. AIC: 478.8951

Results of regression analysis of ΔZ (**longitudinal changes of z-score, n=27**) as a function of selected PCs, using **PVE cutoff=0.8**.

	Estimate	Std.Error	t Value	Pr(>|t|)
(Intercept)	-0.1042	0.9604	-0.108	0.9147
cor.PC4	0.7804	0.3491	2.236	0.0376
sub.PC1	0.6986	0.4497	1.554	0.1368
sub.PC2	0.502	0.3374	1.488	0.1533
sub.PC5	-1.0218	0.3744	-2.729	0.0133
sub.PC6	-1.427	0.4972	-2.87	0.0098
wm.PC1	1.1664	0.46	2.536	0.0202
wm.PC3	2.7464	1.9411	1.415	0.1733

Residual standard error: 1.787 on 19 degrees of freedom

Multiple R-squared: 0.5412. Adjusted R-squared: 0.3722

F-statistic: 3.202 on 7 and 19 DF, p-value: 0.02047. AIC: 116.4815

Results of regression analysis of ΔZ (**longitudinal changes of z-score, n=27**) as a function of selected PCs, using **PVE cutoff=0.9**.

	Estimate	Std. Error	t value	Pr(>|t|)
(Intercept)	3.9443	1.0761	3.665	0.0044
cor.PC1	0.6619	0.4474	1.48	0.1698
cor.PC3	1.6494	0.5332	3.094	0.0114
cor.PC4	0.7519	0.4175	1.801	0.1019
cor.PC7	2.0422	0.8012	2.549	0.0289
sub.PC1	0.4387	0.4009	1.094	0.2994
sub.PC2	-0.3858	0.2639	-1.462	0.1745
sub.PC4	-0.9838	0.3307	-2.975	0.0139
sub.PC5	-1.2007	0.3809	-3.152	0.0103
sub.PC7	-0.6308	0.6096	-1.035	0.3252
sub.PC8	2.2315	0.4353	5.127	0.0004
sub.PC10	-1.4889	0.4392	-3.39	0.0069
sub.PC11	-0.7438	0.5109	-1.456	0.1761
sub.PC12	1.9294	0.6762	2.853	0.0171
wm.PC1	1.3189	0.4461	2.957	0.0144
wm.PC2	-0.6491	0.4491	-1.445	0.1790
wm.PC3	4.1505	1.8511	2.242	0.0488

Residual standard error: 1.365 on 10 degrees of freedom

Multiple R-squared: 0.8592. Adjusted R-squared: **0.6339**

F-statistic: 3.814 on 16 and 10 DF, p-value: 0.01849. AIC: 102.5882

Similar to Model 1, using PVE=0.9 outperforms PVE=0.8 for Model 2. Adopting PVE=0.9, Model 2 attains an adjusted $R^2 = 0.1450$ (p=0.008871) for predicting Z, and 0.6339 (p=0.01849) for predicting ΔZ. Compared with Model 1, Model 2 provides a better fit to the data, demonstrated by consistently higher R^2 values and smaller residual standard errors. Models using PVE=0.8 also achieve better (smaller) AICs than the comparable models using PVE=0.9.

In summary, while there is only a modest association between various imaging markers and baseline cognitive Z-scores (Z), their relationship with longitudinal changes of Z-scores (ΔZ) is notably stronger and more consistent.
